# Outcomes of prophylactic aortocaval stenting before postchemotherapy retroperitoneal lymph node dissection

**DOI:** 10.1016/j.jvscit.2026.102421

**Published:** 2026-07-21

**Authors:** Gowri Gowda, Matthew Vuoncino, Rafael Ricon, Misty D. Humphries

**Affiliations:** Department of Vascular Surgery, University of California, Davis Medical Center, Sacramento, CA

**Keywords:** Hemorrhage, Prophylactic stenting, Retroperitoneal lymph node dissection

## Abstract

Intraoperative hemorrhage during postchemotherapy retroperitoneal lymph node dissection (PC-RPLND) for germ cell tumor resection is a serious concern, typically managed with reactive open techniques involving both vascular and oncologic surgeons. In this study, we reviewed 39 patients who underwent PC-RPLND, including eight who underwent prophylactic aortocaval stenting. Patients with high-risk tumor anatomy who received preoperative stents had significantly lower estimated blood loss compared with nonstented patients (1068.75 ± 835 vs 2528.57 ± 1612 mL; *P* = .043), suggesting that prophylactic aortocaval stenting may be a valuable adjunct to reduce intraoperative bleeding in select high-risk patients who underwent PC-RPLND.

Residual retroperitoneal masses following chemotherapy for germ cell tumors (GCTs) pose a significant risk of morbidity and mortality. Forty percent of retroperitoneal masses can harbor teratomas and 15% may have chemotherapy-resistant GCTs.^1^ Current diagnostic tools are unreliable in distinguishing between teratomas and GCTs, making postchemotherapy retroperitoneal lymph node dissection (PC-RPLND) an integral curative procedure in the treatment of advanced testicular cancer.^2^ PC-RPLND is technically challenging, often complicated by tumor invasion of the major retroperitoneal vessels as seen in Figs 1 and 2. Twenty-five percent of PC-RPLND cases will require additional procedures, including vascular surgery interventions.^3^ Limited literature exists describing endovascular interventions as a pre-emptive adjunct for hemorrhage control during oncologic resection.^4^^,^^5^ A previous study from our institution described preoperative endovascular stenting of the aorta, inferior vena cava (IVC), and/or common iliac veins before oncologic resection as an adjunct for hemorrhage control.^6^ This project aims to further describe prophylactic aorto- and/or caval stenting to reduce intraoperative hemorrhage in patients undergoing RPLND.

**Fig 1:**
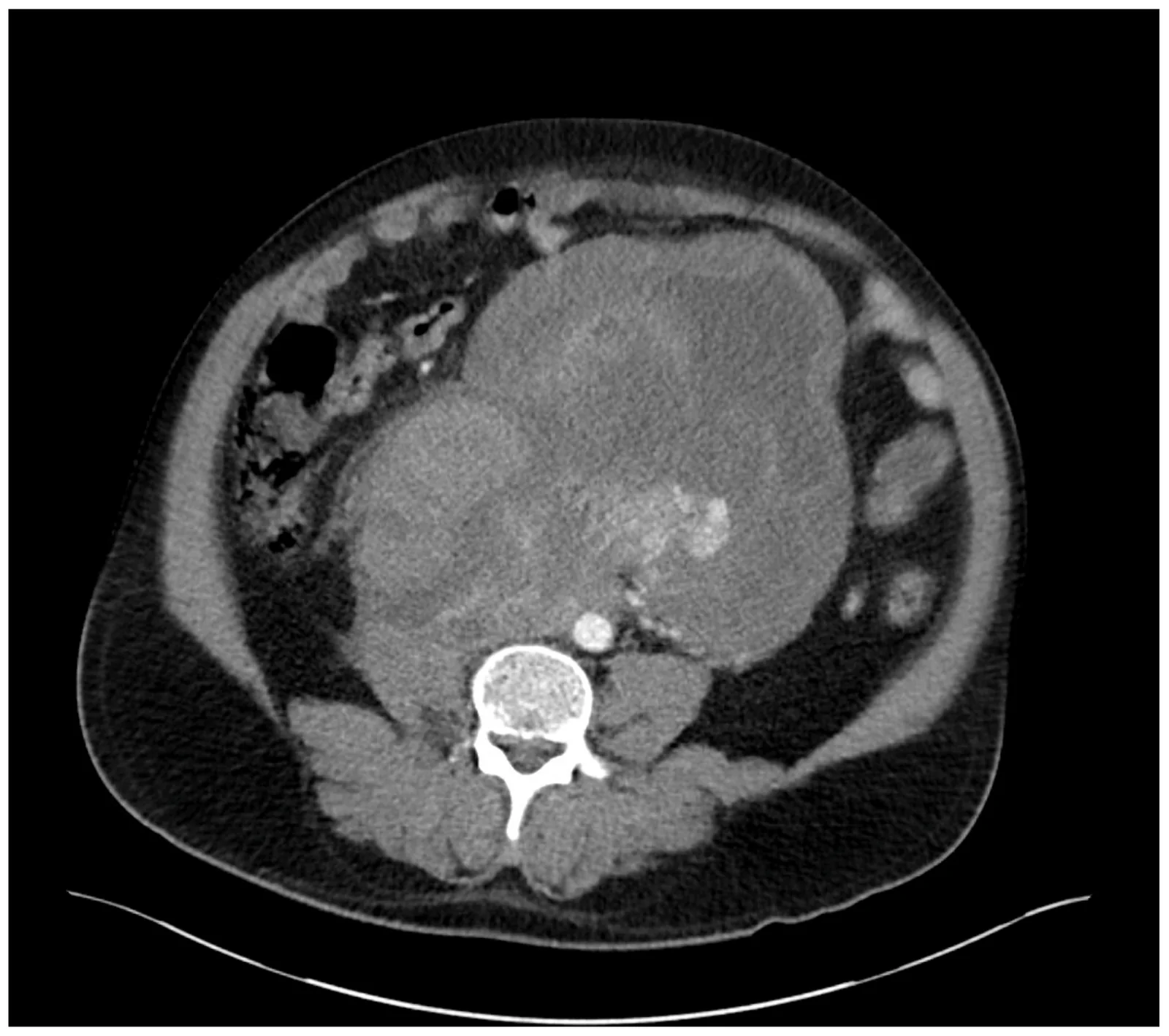
An axial germ cell tumor invasion of the major retroperitoneal vessels.

**Fig 2:**
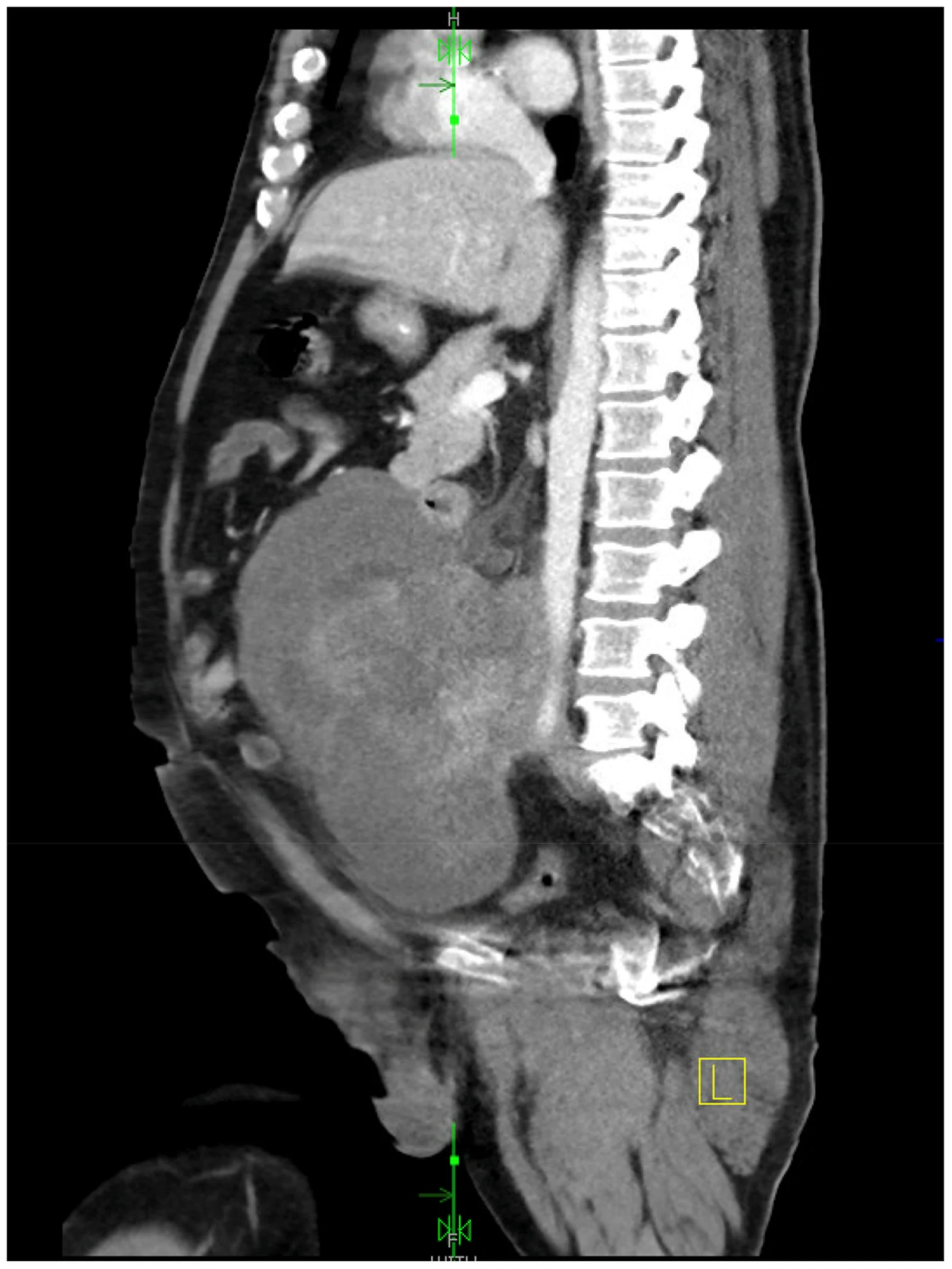
Sagittal germ cell tumor invasion of the major retroperitoneal vessels.

## Methods

A retrospective cohort study of all patients who underwent PC-RPLND at a large tertiary care institution between January 2014 and August 2022 was conducted. Institutional Review Board approval was obtained, and charts were reviewed to determine whether patients underwent angiograms before PC-RPLND. All patients underwent review of preoperative computed tomography scans to stratify their anatomic risk. Patients with high-risk tumor anatomy were defined as those presenting with malignancies encasing greater than 50% of the IVC, aorta, or iliac vessels. Prophylactic stenting was offered to patients meeting this high-risk definition. The electronic medical record was used to review and capture demographic information, 30-day mortality, estimated blood loss, transfusion volume, intensive care unit length of stay, operative time, comorbidities, and anticoagulant use. Statistical analyses were restricted to patients with high-risk tumor anatomy, comparing those with standard operative management (control) and those who underwent endovascular intervention (treatment), using *χ*^2^ test or *t*-test as appropriate.

## Results

Between 2014 and 2022, 39 patients underwent PC-RPLND. Fifteen patients (38%) were deemed to have a high-risk tumor. Nine patients were identified as having angiograms performed before oncologic dissection, eight of whom subsequently underwent staged prophylactic endovascular stenting before PC-RPLND (Table I). The average age of patients who underwent angiography was 34.7 ± 9.14 years, compared with 37.86 ± 11.3 years who did not (*P* = .29). One patient (11%) had no stent placed due to IVC occlusion, two patients (22%) had endovascular coverage of the abdominal aorta, and eight patients (88%) had endovascular coverage of the IVC and/or iliac veins. Venous interventions included self-expanding covered stents (1), aortic cuffs (2), bifurcated aortic devices (3), and thoracic aortic endografts (6) (Table II). Heparin was intraoperatively administered to all nine patients, and the eight patients who had devices implanted were discharged on oral anticoagulation for a minimum 3-month course. No patients who underwent prophylactic stenting had a history of chronic obstructive pulmonary disease, chronic kidney disease, peripheral vascular disease, or myocardial infarction. Average follow-up was 13.1 months, with computed tomography imaging at 1, 6, and 12 months. One stented patient (12.5%) developed phlegmasia cerulea dolens within 30 days requiring thrombectomy, venoplasty, and arteriovenous fistula creation. One stented patient required aortic repair due to tumor involvement of a previous aortic graft; no iatrogenic vascular injuries occurred. The long-term complications included iliac limb thrombosis (n = 1), aortoenteric fistula (n = 1), and fatal graft infection (n = 1).

**Table I:** Risk stratification of study cohort

Tumor-risk categories	Entire cohort (N = 39)	Prophylactic stenting (N = 8)	No prophylactic stenting (N = 31)
High-risk anatomy^a^	15 (38)	8 (100)	7 (23)
Standard-risk anatomy	24 (62)	0 (0)	24 (77)

Categorical data are shown as number (%).

a High-risk anatomy was defined as malignancies encasing >50% of the inferior vena cava, aorta, or iliac vessels on preoperative computed tomography (CT) imaging.

**Table II:** Prophylactic interventions

Age, years	Gender	Caval intervention	Aortic intervention
48	M	Gore CTAG	Gore Cuff
37	M	None, thrombosed IVC	-
20	M	Gore CTAG	-
26	M	Gore CTAG + Gore Excluder	-
48	M	Gore CTAG + VIABAHN × 2	-
27	M	Gore Cuff + Gore Excluder	-
40	M	CTAG + Gore Excluder	-
35	M	Gore CTAG	-
31	M	Gore cuff × 2	Gore Excluder

*IVC*, Inferior vena cava.

Average operative time was 95.4 ± 30.3 minutes for stenting and 300.8 ± 71.8 minutes for oncologic resection. The average PC-RPLND operative times for high-risk patients was similar between treatment and control groups, 286.9 vs 301.7 minutes (*P* = .72). The average intensive care unit length of stay between groups was similar, 5.4 vs 3.9 days (*P* = .33). Average estimated blood loss was significantly higher in the control group compared with the treatment group, 2.5 vs 1.0 L, respectively (*P* = .04). The incidence of transfusion in high-risk patients was similar between the treatment and control groups, 0.7 vs 0.6 (*P* = .31), with similar transfusion volumes, 762.5 vs 1739 mL, respectively (*P* = .06) (Table III). Thirty-day mortality was zero in both groups.

**Table III:** Outcomes among patients with high-risk tumor anatomy undergoing prophylactic stenting vs standard management

Variables	M or proportion	SD	M or proportion	SD	*P* value
Thirty-day mortality	0	0	0	0	
Operative time, min	286.857	75.16	301.75	81.34	.72
EBL, mL	2528.57	1612.16	1068.75	835.779	**.043**^a^
Transfusion volume, mL	1738.6	1121.92	762.5	425.892	.065
Transfusion incidence (proportion)	0.714		0.625		.313
ICU LOS, days	0.857	1.215	3.857	3.132	.326
Antiplatelet/anticoagulant use	0.43		0.88		.067

*EBL*, Estimated blood loss; *ICU*, intensive care unit; *LOS*, length of stay; *SD*, standard deviation.

Boldface *P* value represents statistically significant difference.

a Denotes statistically significant difference.

## Discussion

Prophylactic endovascular interventions offer a minimally invasive approach to mitigating vascular challenges during PC-RPLND for GCTs. An initial case series proposed this method as safe and viable.^9^ Of 15 high-risk patients, eight underwent prophylactic stenting, six had standard management, and one was not a stent candidate due to chronic IVC occlusion. Previous studies have shown increased transfusion requirements correlated with higher complication rates.^1^ Prophylactic stenting significantly reduced intraoperative blood loss, though transfusion volumes were nonstatistically lower likely due to limited sample size. Nevertheless, we believe endograft placement facilitates more aggressive lymph node dissection, while minimizing the risk of hemorrhage. Although endovascular stent grafts may theoretically complicate lumbar artery back-bleeding during resection, operative reports in our stented cohort revealed no clinically significant back-bleeding attributable to stent presence, with hemostasis achieved via suture ligation and surgical clips.

The traditional alternative to address intraoperative hemorrhage involves open vascular repair, historically required in 1.5% to 10% of cases.^2^^,^^7^^,^^8^ In our cohort, 20% underwent prophylactic stenting; however, from open vascular reconstruction during oncologic resections are notably high. For example, a 2006 case series of open caval and aortic reconstructions in conjunction with retroperitoneal oncologic resections reported an 18% acute graft reintervention rate and a 27% incidence of graft thrombosis beyond 30 days.^9^ Although promising, endovascular interventions carry risks including fracture, migration, maldeployment, and thrombosis. In our cohort, 12.5% experienced acute complications requiring thrombectomy with three long-term complications. Of note, not all patients undergoing PC-RPLND will require vascular intervention. Forty percent of patients in the high-risk control group required no vascular intervention, highlighting patient selection challenges.

As this was a retrospective study, no standardized protocol existed for prophylactic stent placement. Selection for angiography and endovascular intervention was based on multidisciplinary review and surgeon judgment, introducing potential selection bias.

Several limitations should be considered when interpreting these findings. The retrospective nature and small cohort size of our study limit its generalizability. Although stratifying patients into a high anatomic risk group aided in comparison, it does not fully characterize the anatomic burden of disease. The impact of endovascular stenting on ease of dissection and resection remains difficult to quantify, particularly since detailed operative note review was only able to be performed for the stented cohort. Operative time and transfusion requirements serve as difficulty surrogates but may be confounded by patient, institutional, and surgeon factors. Selection bias was an additional limitation in this study. Further studies with larger cohorts are needed to determine the generalizability and investigate the statistical significance of the outcomes observed in this study.

## Conclusions

Prophylactic aortocaval endovascular stenting may significantly reduce intraoperative hemorrhage during PC-RPLND in high-risk patients with GCTs. Future prospective studies with larger cohorts will help define optimal patient selection criteria and long-term outcomes.

## Funding

No funding was provided.

## Disclosures

The authors have no competing interest.
